# Targeting deubiquitinating enzyme USP26 by microRNA-203 regulates Snail1’s pro-metastatic functions in esophageal cancer

**DOI:** 10.1186/s12935-020-01441-2

**Published:** 2020-07-30

**Authors:** Gang Li, Hong-wei Qi, He-gui Dong, Ping Bai, Ming Sun, Hai-yan Liu

**Affiliations:** 1 Department of Surgical Oncology, Taian City Central Hospital, Taian, 271000 Shandong China; 2 Department of Medicine, Taian City Central Hospital, Taian, 271000 Shandong China; 3 Department of Outpatient, Taian City Central Hospital, Taian, 271000 Shandong China; 4 Department of Oncology, The Second Affiliate Hospital of Shandong First Medical University, No.706, Taishan Street, Taian, 271000 Shandong China

**Keywords:** Esophageal cancer, USP26, Snail1, miR-203, Metastasis

## Abstract

**Background:**

Esophageal cancer is one of the most common cancers worldwide with poor prognosis and high mortality. The transcription factor *SNAI1*, encoding Snail1, is important for metastatic progression in esophageal cancer whereas the microRNA (miRNA)-203 has been shown to function as an inhibitor of metastasis in EC. The Snail1 protein is stabilized in EC partially by the deubiquitinating enzyme USP26; however, how USP26 is regulated is not completely known.

**Methods:**

Expression of *SNAI1* and *USP26* messenger RNA (mRNA) and miR-203 was performed in datasets within The Cancer Genome Atlas and Gene Expression Omnibus, respectively. Expression of Snail1 and USP26 protein and miR-203 was determined in the normal esophageal cell line HET-1A and EC cell lines Kyse150 and TE-1 using western blot and quantitative polymerase chain reaction, respectively. TargetScan was used for in situ prediction of miR-203 targets and in vitro heterologous reporter assays using the wild-type and miR-203 seed mutant of the 3′ Untranslated region (UTR) of *USP26* were used to investigate whether *USP26* is a target of miR-203. Effects of increasing miR-203 using MIR203A/5P mimic on USP26 and Snail1 in the HET-1A, Kyse150 and TE-1 cell lines were performed using western blot and cycloheximide-based protein stability analysis. Effects of modulating miR-203 in Kyse150 and TE-1 cell lines on in vitro pro-metastatic effects were analyzed by invasion assay, scratch wound-healing assay, and chemosensitivity to 5-fluoruracil (5-FU). In vivo lung metastasis assay was used to study the effect of modulating miR-203 in Kyse150 cells.

**Results:**

*SNAI1* mRNA and *HSA/MIR203* was higher and lower, respectively, in EC patients compared to tumor-adjacent normal tissues. No changes in expression of *USP26* mRNA were observed in these datasets. *MIR/203* expression was downregulated whereas protein expression of both Snail1 and USP26 were higher in EC cell lines Kyse150 and TE-1 compared to normal esophageal cell line HET-1A. *USP26* was predicted as a potential target of miR-203 by TargetScan Release 2.0. Reporter assays confirmed *USP26* as a target of miR-203 in the EC cell lines. Transfection of EC cell lines with *MIR203* mimic decreased USP26 protein expression and Snail1 protein stability indicating the ability of miR-203 to regulate Snail1 protein levels via USP26. Exogenous increase in miR-203 in the EC cell lines significantly inhibited Snail-1 mediated in vitro pro-metastatic function of invasion, wound-healing, and increased chemosensitivity to 5-FU. Finally, overexpression of miR-203 inhibited in vivo lung metastasis of Kyse150 cells, which was reversed following overexpression of USP26, indicating a direct role of miR-203-mediated regulation of USP26 in metastatic progression of EC.

**Conclusions:**

Cumulatively, these results establish an important mechanism by which decrease in miR-203 expression potentiates metastatic progression in EC via USP26-mediated stabilization of Snail1. Hence, miR-203 can serve as a biomarker of metastasis in EC and is a potential target for therapeutic intervention in EC.

## Background

Esophageal cancer (EC) is the sixth-most common cancer worldwide with high incidence of metastasis detection at initial diagnosis [1–4] and a poor 5-year survival period of 15–25% [5, 6]. Indeed, cancer metastasis is the prime reason for all cancer related mortality, accounting for approximately 90% of deaths associated with cancer [1].

One of the important initiating steps of cancer metastasis is epithelial to mesenchymal transition (EMT), whereby epithelial cancer cells within a primary tumor acquire morphological, phenotypical, and functional changes that aid them to migrate to secondary sites [7]. EMT is also critically important in rendering chemoresistance to cancer cells [7]. Given the importance of EMT in cancer progression it is not surprising that the process is regulated by multiple mechanisms at different transcriptional, post-transcriptional and post-translational stages.

One of the most important and well-characterized transcription factors required for EMT progression is *SNAI1*, which encodes Snail1. Expression of Snail1 was found to be positively correlated to metastatic progression in EC patients [8, 9]. Snail1 protein is post-translationally regulated by the ubiquitin-proteasome system, with SPSB3, FBXO11, FBXL14 and β-TrCP functioning as the cognate E3 ligases in a context-dependent fashion [10–13]. These actions of E3 ligases have been shown to be regulated among others by deubiquitinating enzymes (DUBs), including DUB3, OTUB1, PSMD14, and USP26 [14–18]. USP26-mediated regulation of Snail1 stabilization has been shown to potentiate metastatic progression in EC [18]. However, how expression of USP26 is regulated is not currently known.

Much like the protein effectors of EMT and metastatic progression, several microRNAs (miRNAs) have been indicated to be critical in EMT and metastatic progression [19–23]. Among the different miRNAs that have been shown to regulate different steps of EC, there seems to be a consensus about the miR-203 functioning as an inhibitor of metastatic progression in EC [24] with its expression downregulated in EC. It has been reported that miR-203 inhibits EC progression by targeting the stem cell renewal factor Bmi-1 [25]. However, whether there are additional targets of miR-203 that potentiates its role in suppressing EC is not known.

In this study we confirm that miR-203 expression is significantly downregulated in both EC patients and cell lines compared to tumor-adjacent normal tissue and normal human esophageal squamous cell line, respectively. *USP26* was found to be a putative target of miR-203 and was confirmed as a bona fide target by a combination of heterologous reporter assays and in vitro and *in vivo* functional studies. Our results also showed that exogenous modulation of USP26 expression in EC cell lines by miR-203 directly impact Snail1 protein stability and pro-metastatic in vitro functions of migration, invasion, and chemoresistance to 5-fluorouracil (5-FU), as well as in vivo metastasis. These results establish miR-203 as a potential biomarker as well as an attractive therapeutic target in EC.

## Methods

### Cell culture

The normal esophagus cell line HET-1A and EC cell lines, Kyse150 and TE-1 were purchased from ATCC (USA) and iCell Bioscience Inc., Shanghai, China, respectively. HET-1A was cultured in BEGM (BEBM along with additives except for the gentamycin-amphotericin B mix) (Lonza Clonoletics Corporation, USA). TE-1 and Kyse150 cells were cultured in 10% fetal bovine serum (Thermo Fisher Scientific, USA) containing RPMI1640 (Thermo Fisher Scientific) media. All cells were maintained in an incubator at 37  °C, saturated humidity, and 5% carbon dioxide. For Snail1 protein stability assays, cells were treated with cycloheximide (50 µg/ml; Sigma-Aldrich, USA) for up to 8 h.

### Immunoblot analysis

At the end of experimental time points cells were washed with ice-cold 1X phosphate buffered saline and then lysed using RIPA buffer (Thermo Fisher Scientific). Protein concentrations were determined by BCA kit (Thermo Fisher Scientific). Fifty micrograms of protein lysates were resolved by 10% sodium dodecyl sulfate polyacrylamide gel electrophoresis and then transferred to PVDF membranes. Membranes were blotted using the following antibodies as indicated: SNAIL1 (clone 20C8, 1:500, Thermo Fisher Scientific), USP26 (catalogue # PA5-96893, 1:1000, Thermo Fisher Scientific), E-cadherin (catalogue # 3195, 1:1000; Cell Signaling Technologies, USA)and GAPDH (catalogue # MA5-15738, 1:5000, Thermo Fisher Scientific). GAPDH was used as a loading control. All immunoblot images are representative of 3 experiments.

### Isolation of miRNA and quantitative real time polymerase chain reaction (qRT-PCR)

At the end of experimental time points cells were washed with ice-cold 1× phosphate buffered saline. Cell pellets were then used to isolate miRNA using PureLink miRNA isolation kit (Thermo Fisher Scientific). qRT-PCR was done using TaqMan probes (hsa-miR-203a-5p: assay ID-477013_mat and *RNU6B*: assay ID-001093; Thermo Fisher Scientific). Expression of miR-203a-5p was normalized to *RNU6B* expression and relative expression in HET-1A, Kyse150, and TE-1 cells lines were calculated using the 2^− ΔΔCt^ method. Data was presented as scattered dot plot (mean ± standard deviation (SD)) of six biological replicates, each done in three technical replicates.

### Bioinformatic analysis

cBioPortal (https://www.cbioportal.org/) was used to analyze the relative expression of *SNAI1* and *USP26* in 1687 patients. Non-coding RNA profiling array data were downloaded from GEO [26]—GSE. TargetScan Human release 2.0 was used to predict potential targets of miR-203a-5p and potential miRNAs binding 3′UTR of *USP26*. Our analysis revealed that *USP26* 3′UTR was found to harbor a 7mer-A8 seed for miR-203a-5p.

### Plasmids, mimics, and transfection

The wild-type 3′UTR of *USP26* was amplified from genomic DNA using forward primer 5′-ctccttgtacagatctgcctga-3′ and reverse primer 5′-tcacaaaggcaaggcataca-3′ and cloned into pGL3 vector (Promega, USA). The miR-203a-5p seed mutant 3′UTR of *USP26* (mutant 3′UTR) was generated by site directed mutagenesis using Quick Change II kit (Agilent, USA) and the following primers: 5′-tgtagtacagtagttgctctcaaactgtatcaagcatcacggtc-3′ and 5′-gaccgtgatgcttgatacagtttgagagcaactactgtactaca-3′. The miR-203a-5p (MIR203A/5P) and non-sense scrambled negative control (control) mimics were purchased from Thermo Fisher Scientific. For reporter assays, cells (4 × 10^4^) were plated in 24-well plates 24 h before transfection. Cells were transfected with 0.5 µg each of 3′UTR containing pGL3 vector and a control pSV40-RLL (encoding Renilla luciferase, internal control) using Lipofectamine 3000 (Thermo Fisher Scientific). Where indicated, cells were transfected with 30 nM of control or MIR203A/5P mimic using RNAiMAX reagent (Thermo Fisher Scientific) 24 h before transfection of the luciferase reporter plasmids. Reporter assays were performed 24 h after transfection using Dual Luciferase Assay kit (Promega). Firefly luciferase values were normalized using Renilla luciferase values from the same wells and then relative changes in reporter activities were calculated for each experimental setting. Data was plotted as scattered dot plot (median ± range) of three biological replicates. For generating cells for xenograft assay, Kyse150 cells were transduced using lentivirus encoding Firefly luciferase and selected using G418 (500 µg/ml, Thermo Fisher Scientific) for 2 weeks. Firefly luciferase expressing Kyse150 cells were subsequently transduced with *USP26* expressing lentivirus (#RC223359L1V, Origene) and selected using puromycin (2 µg/ml, Thermo Fisher Scientific) for 2 weeks.

### Invasion assay

In vitro invasion assay was performed in 96-well format using Cultrex Basement Membrane Extract Cell Invasion Assay kit (catalog # 3455-096-K; R&D Systems, USA). Cells (5 × 10^4^) were serum starved for 12 h before adding to the top chambers, whereas complete medium was used as chemoattractant in all cases. Post-experiment bottom of wells was stained using crystal violet and imaged. Quantification was done by lysing and measuring fluorescent activity using Calcein AM as per manufacturer’s protocol. Absolute number of cells were calculated from standard curve using OD values obtained from Calcein AM assay. Data was plotted as scattered dot plot (median ± range) of three biological replicates, each done in duplicate technical controls.

### Migration–Scratch wound-healing assay

Twenty-four hours after transfection with control of MIR203A/5P mimic, TE-1 and Kyse150 cells were platted in six-well flat-bottom plates (Thermo Fisher Scientific) and allowed to grow to a confluent cell monolayer. Scratching was performed using a 200-µl sterile pipette tip. Subsequently, the wells were washed with 1 × PBS to get rid of the floating cells. The cells were cultured in growth medium and imaged 24 h later. Migration was defined as percentage of wound closure after 24 h and calculated as: [(Original gap distance − Gap distance after 24 h) / (Original gap distance)] x 100.

### Chemosensitivity assay

Kyse150 and TE-1 cells were transfected with control or *MIR203A/5P* mimic for 24 h as described above before being treated with indicated doses of 5-FU (Sigma-Aldrich) for 24 h. After treatment period, relative cell viability was quantified using MTT assay (Sigma-Aldrich) as per manufacturer’s protocol. Data was plotted as staggered representation of each replicate.

### Mouse models of metastasis and tissue processing

Institutional Animal Care and Use Committees of The Second Affiliate Hospital of Shandong First Medical University approved all animal studies conducted in the context of the current study. BALB/c nude mice were intravenously injected via lateral tail vein with parental Kyse50 cells stably expressing firefly luciferase (n = 10) or Kyse150 cells stably expressing firefly luciferase and *USP26* (n = 05) (1 × 10^6^ in 100 µl PBS). Twenty-four hours later mice injected with parental Kyse150 cells were randomly divided into 2 groups – control and MIR203A/5P mimic group. Mice in the control group were injected with 100 mg/kg control mimic every alternate day, whereas mice in the MIR203A/5P mimic group were injected with 100 mg/kg MIR203A/5P mimic every alternate day. Mice injected with Kyse150 cells overexpressing *USP26* were also injected with 100 mg/kg MIR203A/5P mimic every alternate day. Bioluminescence imaging for firefly luciferase was used to track the experimental metastatic progression. Animals were euthanized in humane fashion after 6 weeks and lungs were excised and fixed overnight in 10% neutral buffered formalin. Tissue specimens were processed using routine procedures for H&E staining and evaluated for histological assessment of lung metastasis. Immunohistochemistry for USP26 was done using USP26 antibody (catalogue # PA5-96893, 1:50, Thermo Fisher Scientific) following manufacturer’s protocol.

### Statistical analysis

All data were analyzed using GraphPad Prism V8. Statistical difference between groups was calculated using unpaired non-parametric Mann Whitney test. P < 0.05 was considered as statistically significant.

## Results

### Differential regulation of SNAI1 and hsa-miR-203 in esophageal cancer patient samples

**Fig. 1:**
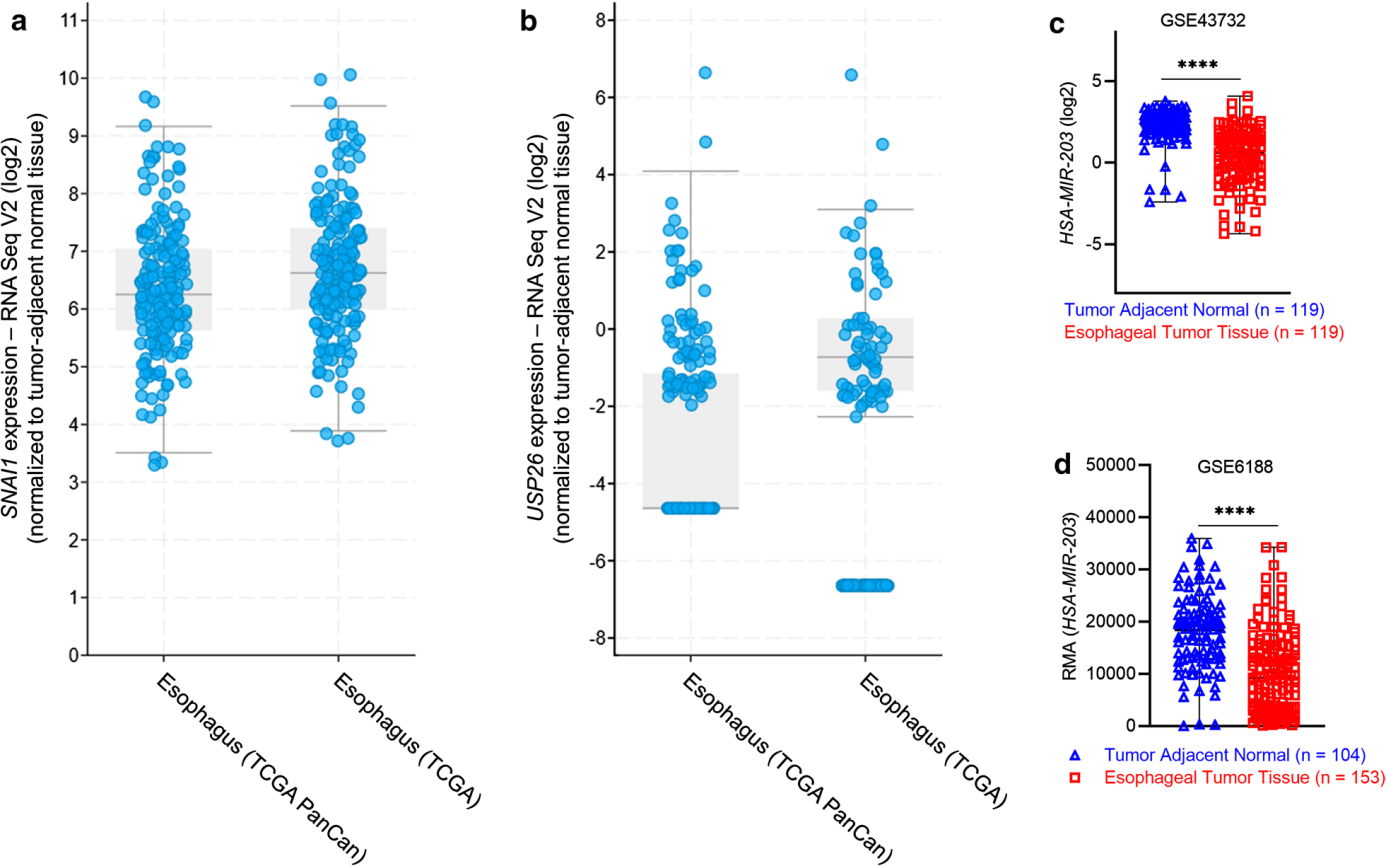
SNAI1 is upregulated and hsa-miR-203 is downregulated in esophageal cancer.  Fold-change (log2) of *SNAI1* (**a**) and *USP26* (**b**) in esophageal cancer tumor tissue compared to tumor adjacent normal tissues. Data in **a** and **b** are from 1687 patients from TCGA analyzed by the cBioPortal. **c**, **d** Expression profile of miR-203 analyzed from non-coding RNA profiling array data downloaded from GEO by GEO2R. Shown are log2 converted expression in 119 paired samples obtained from GSE43732 (**c**), robust multiarray analysis (RMA) raw values in 104 tumor-adjacent normal tissue and 153 esophageal tumor tissue in GSE6188. (Fig. 1d; P < 0.0001). Shown are scatter plots, mean with range; ****P < 0.0001

It has been shown that *SNAI1* expression is correlated to EC progression [9] and USP26 stabilizes Snail1 protein in EC [18]. Hence, we initially confirmed expression of *SNAI1* and *USP26* messenger RNA (mRNA) in EC patients. *SNAI1* was upregulated 6.23 ± 3.11 folds in the Esophagus (TCGA PanCan) data set and 6.59 ± 3.02 folds in the Esophagus (TCGA) datasets, respectively (Fig. 1a). There was no significant change in expression of *USP26* in the same datasets (Fig. 1b). We next analyzed miR-203 expression in GSE43732 and GSE6188. It is important to note that previous analysis of these 2 datasets by He et al. 2019 [24] had shown miR-203 as one of 17 differentially expressed miRNA between EC and tumor-adjacent normal tissue in patient samples. The goal of our re-analysis was to exclusively represent relative expression of miR-203 in EC tissue and tumor-adjacent normal tissue. Confirming the previous findings [24], in 119 paired patient samples in GSE43732, miR-203 expression was significantly downregulated in esophageal tumor tissue (Fig. 1c; P < 0.0001) and in 104 tumor-adjacent normal tissue and 153 esophageal tumor tissue patient samples in GSE6188, miR-203 expression was significantly downregulated in esophageal tumor tissue (Fig. 1d; P < 0.0001). Overall, these results validated that *SNAI1* and miR-203 expressions are up and down regulated, respectively, in EC.

### **Differential expression of Snail1 and USP26 protein and miR-203a-5p in normal esophagus and EC cell lines**

**Fig. 2:**
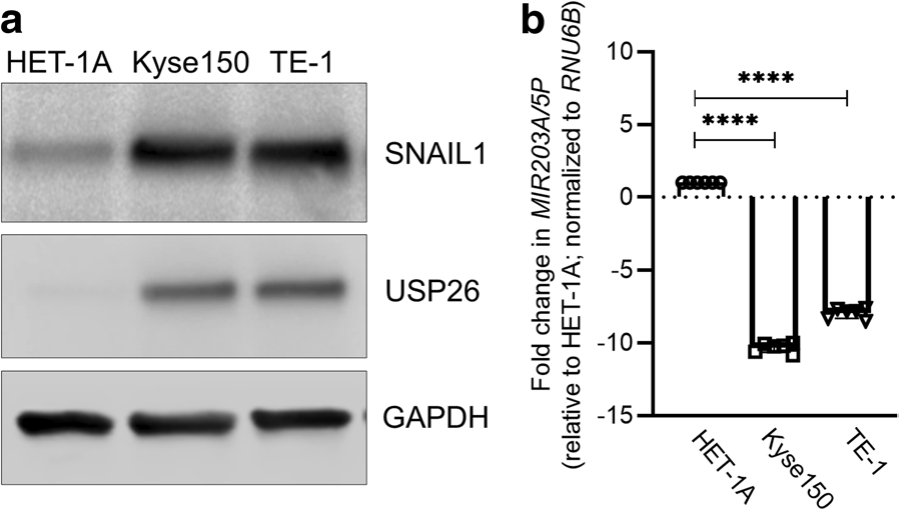
Differential expression of Snail1 and USP26 protein and miR-203a-5p in normal esophagus and EC cell lines. **a** Representative blots from three experiments showing steady state expression of Snail1 and USP26 in the normal esophageal cell line HET-2A and the EC cell lines Kyse150 and TE-1. GAPDH. Loading control. **b** Relative expression of miR-203-5p in the three cell lines determined by qRT-PCR. Shown are scattered plot, mean ± SD (n = 06). ****P < 0.0001

We next performed expression analysis in the normal esophagus cell line HET-1A and the EC cell lines Kyse150 and TE-1. Steady state protein expression of both Snail1 and USP26 were higher in the EC cell lines compared to the HET-1A cell line, where USP26 was hardly detected (Fig. 2a). Compared to HET-1A, expression of miR-203 was 10.31 ± 0.33 folds and 7.36 ± 0.88 folds downregulated in Kyse150 and TE-1 cells, respectively (Fig. 2b; P < 0.0001 in each case compared to HET-1A).

### ***USP26*** is a target of miR-203 in esophageal cancer cell lines

**Fig. 3:**
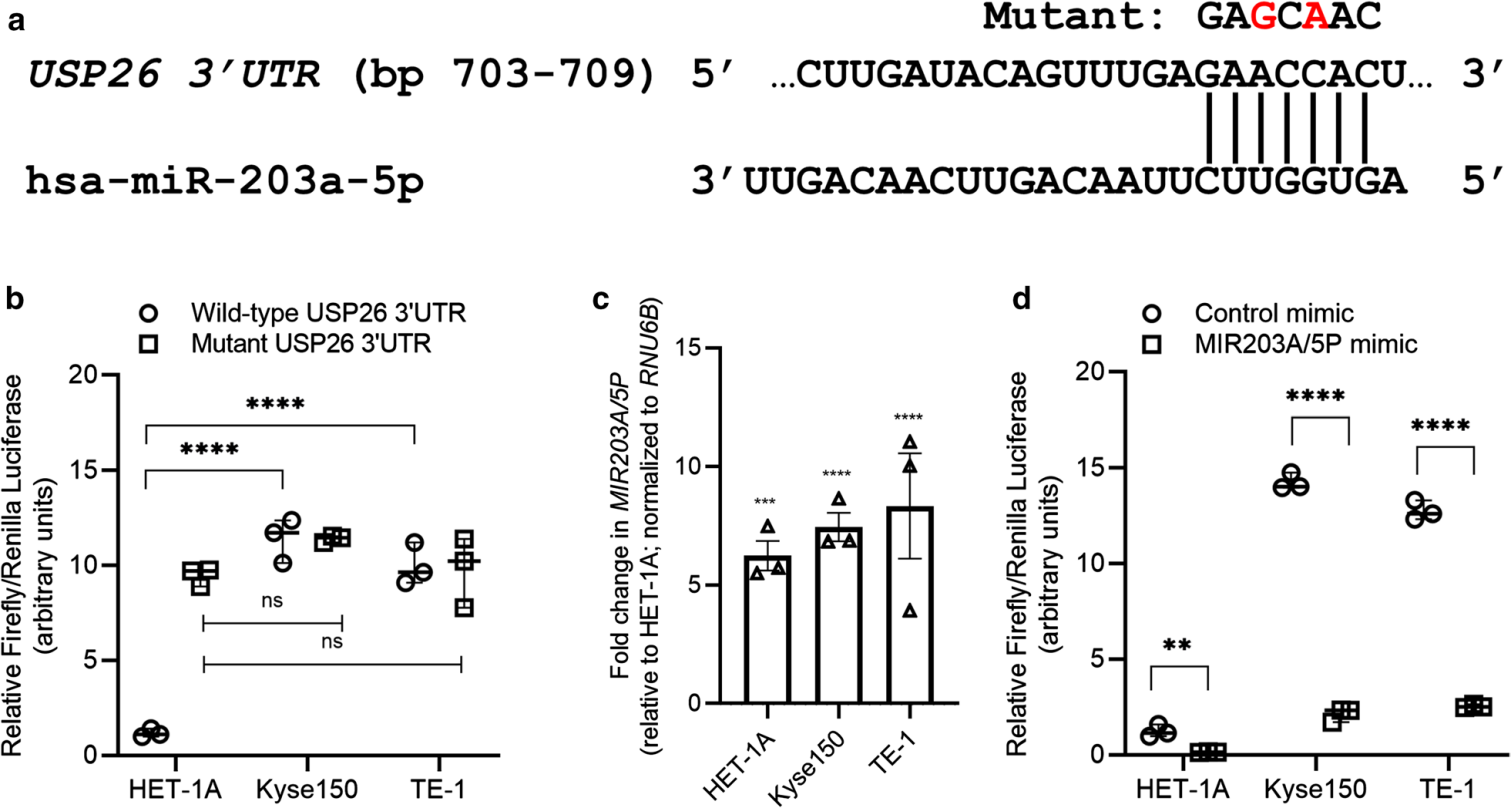
*USP26* is targeted by miR-203. **a** Schematic representing complementary sequences in the 3′ untranslated region (UTR) of *USP26* and seed sequence of miR-203a-5p. On top is shown the bases in red that were mutated to generate the mutant heterologous reporter construct. **b** Relative reporter activity (firefly luciferase normalized to renilla luciferase) in HET-1A, Kyse150, and TE-1 cells transfected with wild-type or mutant *USP26* 3′UTR reporter construct. Shown are scattered plot, median with range (n = 03). **c** Relative expression of miR-203-5p in the three cell lines transfected with control or MIR203A/5P mimic as determined by qRT-PCR. Shown are scattered plot, mean ± SD (n = 03). **d** Relative reporter activity (firefly luciferase normalized to renilla luciferase) in HET-1A, Kyse150, and TE-1 cells transfected with wild-type *USP26* 3′UTR reporter construct along with control or MIR203A/5P mimic. Shown are scattered plot, median with range (n = 03). **P < 0.01; ***P = 0.001; ****P < 0.00001; ns, not significant

Given that *USP26* mRNA was not changing between normal and tumor esophagus tissue but were increasing at the protein level, we hypothesized that *USP26* is regulated by miRNA at the level of mRNA translation. We used TargetScan7 v2.0 to predict putative miRNAs targeting the 3′UTR of *USP26*. Given that miR-203a-5p was downregulated in EC, we also used TargetScan7 v2.0 to predict putative gene targets of miR-203a-5p. Both analyses revealed *USP26* was a putative target of miR-203a-5p with *USP26* harboring a 7merm8 seed for miR-203a-5p (Fig. 3a).

In order to confirm if *USP26* is a real target of miR-203a-5p we generated firefly luciferase reporter plasmid harboring either the wild-type or miR-203a-5p seed mutant 3′UTR of *USP26*. Luciferase reporter assay showed that reporter activity of the wild-type 3′UTR in HET-1A cells was repressed 10.24 ± 1.09 and 8.81 ± 1.11 folds compared to Kyse150 and TE-1 cells, respectively (Fig. 3b; P < 0.0001 in each case). This observation was in sync with significantly lower expression of miR-203A-5p in the Kyse150 and TE-1 cells compared to HET-1A cells (Fig. 2b). If indeed the difference in reporter activity was due to difference in expression of miR-203a-5p then mutating the binding site for miR-203a-5p in the reporters would attenuate the repression observed in the HET-1A cells. Indeed, when mutant reporters were used no significant differences in reporter activity were observed in the HET-1A, Kyse150 and TE-1 cells (Fig. 2b). For further convincing evidence, all three cells were transfected with either a control or MIR203A/5P mimic. Successful transfection was verified by qRT-PCR (Fig. 3c). These cells were subsequently transfected with the wild type reporter constructs and relative reporter activity in the cells transfected with control or MIR203A/5P mimic were compared. Transfection of MIR203A/5P mimic significantly decreased reporter activity by 8.56 ± 1.61 (P < 0.01), 6.84 ± 1.17 (P < 0.0001), and 5.02 ± 0.26 (P < 0.001) folds in the HET-1A, Kyse150, and TE-1 cells, respectively (Fig. 3d). These results confirmed that *USP26* is truly being targeted by miR-203a-5P in the normal esophagus cell line HET-1A and that it is derepressed in the EC cell lines TE-1 and Kyse150.

### Modulating miR-203 expression in EC cell lines impact stability of Snail1 protein

**Fig. 4:**
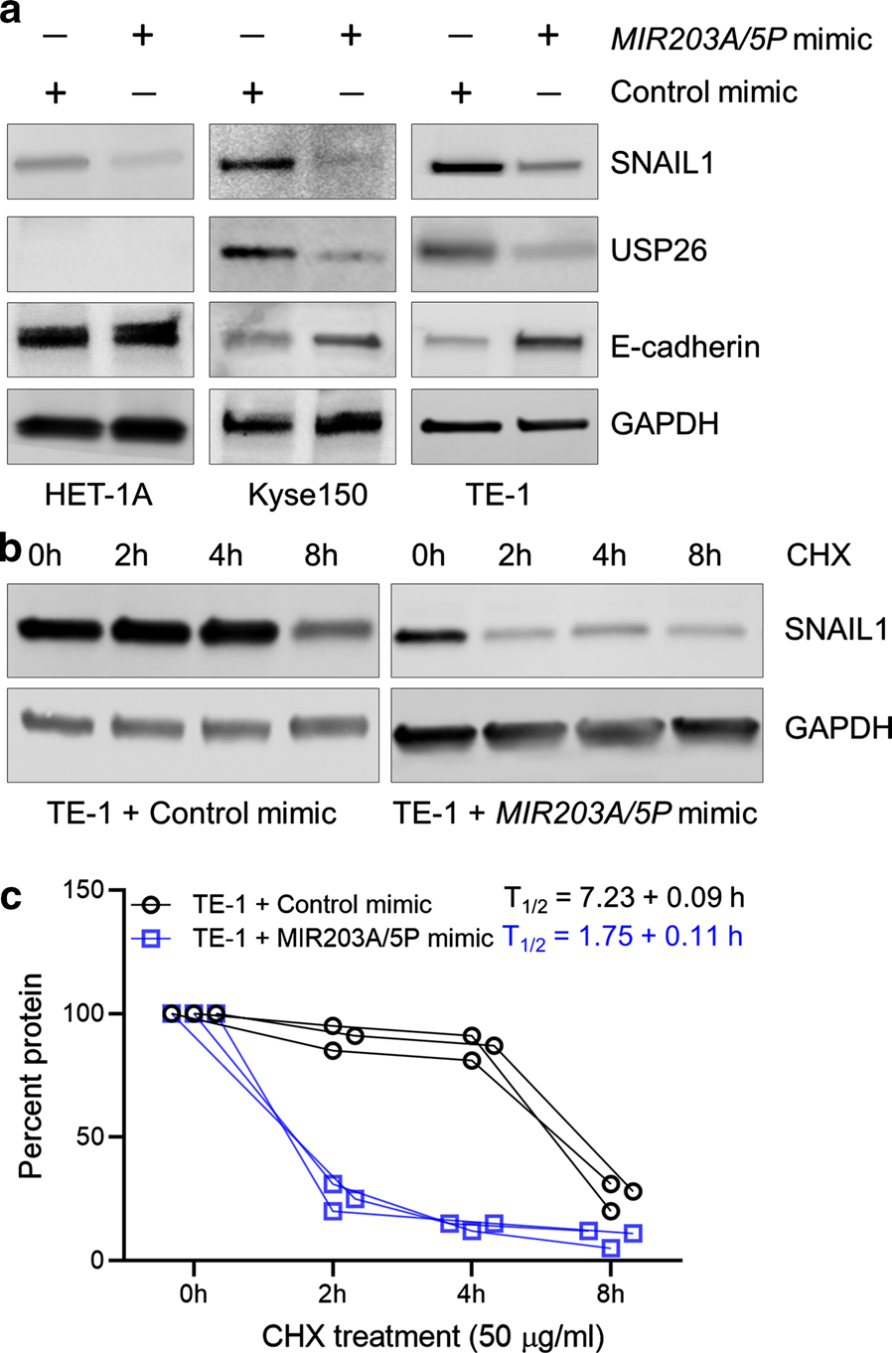
Modulating miR-203 expression in EC cell lines impact Snail1 protein stability. **a** Representative blots from three experiments showing steady expression of Snail1, USP26 and E-cadherin in the normal esophageal cell line HET-1A and the EC cell lines Kyse150 and TE-1 transfected with either control or MIR203A/5P mimic. GAPDH. Loading control. **b** TE-1 cells transfected with control or MIR203A/5P mimic were treated with 50 µM cycloheximide (CHX) for up to 8 h and then lysates were probed for Snail1 and GAPDH protein expression. Shown are representative blots from three experiments. **c** Blots in **b** were analyzed using NIH ImageJ algorithm and half-life of Snail1 protein calculated

If *USP26* was indeed being targeted by miR-203a-5P, then it would result in downregulation of USP26 protein expression and by extension destabilize Snail1 protein. To investigate that immunoblot analysis was performed in the HET-1A, Kyse150, and TE-1 cells transfected with control or MIR203A/5P mimic. The MIR203A/5P mimic caused a robust downregulation of both USP26 and Snail1 in both the Kyse150 and TE-1 cells (Fig. 4a). Given Snail1 function in EMT by downregulating the epithelial cell marker E-cadherin [9], we also determined expression of E-cadherin in the control or MIR203A/5P mimic-transfected HET-1A, Kyse150, and TE-1 cell lines. The MIR203A/5P mimic increased E-cadherin in both the Kyse150 and TE-1 cells (Fig. 4a).

To confirm that the change in Snail1 protein expression in the MIR203A/5P mimic-transfected cells was due to decrease in Snail1 stability caused by a decrease in USP26 protein, we performed protein stability analysis in TE-1 cells transfected with control or MIR203A/5P mimic. Cells were treated with the translation elongation inhibitor cycloheximide for up to 8 h and relative expression of Snail1 protein was determined by immunoblot analysis. As shown in Fig. 4b, Snail1 protein was lower at 0 h in the MIR203A/5P mimic transfected TE-1 cells compared to the control mimic transected cells. These blots also suggested that Snail1 protein was degrading faster in the MIR203A/5P mimic transfected TE-1 cells. The half-life of Snail1 protein was significantly lowered following MIR203A/5P mimic transfection in the TE-1 cells 1.75 ± 0.11 h compared to 7.23 ± 0.09 h in the control mimic transfected cells (Fig. 4c; P > 0.0001). These results proved that the decrease in Snail1 protein following upregulation of miR-203a-5p upregulation is due to increased degradation of the Snail1 protein induced by a loss of USP26 protein expression.

### Overexpression of miR-203 in EC cell lines inhibit in vitro pro-metastatic functions

**Fig. 5:**
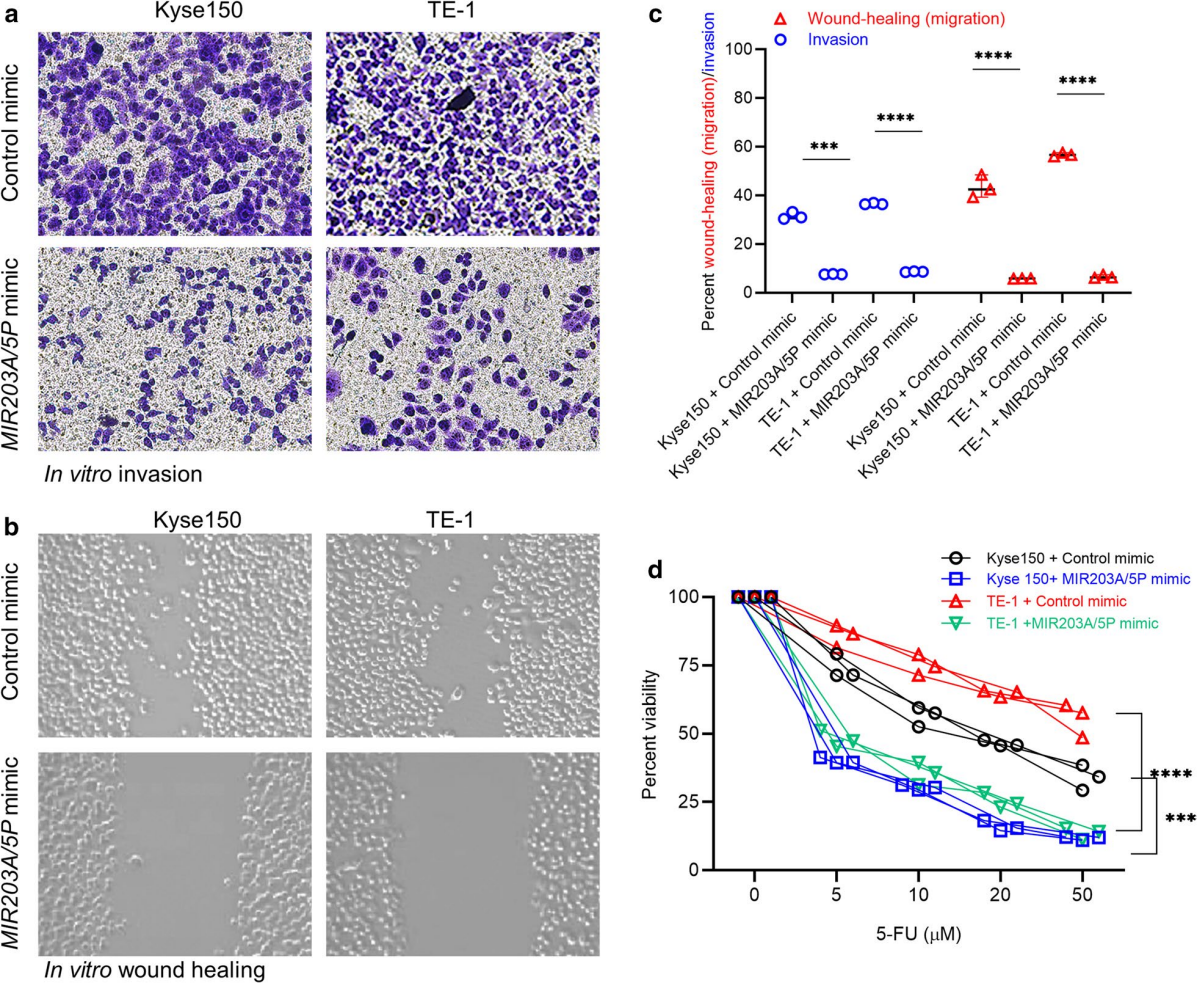
Modulating miR-203 expression in EC cells impact ***in vitro*** pro-metastatic functions of Snail1 protein. **a**, **b** Representative photomicrograph images of *in vitro* invasion (**a**) and migration **(b**) of Kyse150 and TE-1 cells transfected with either a control or MIR203A/5P mimic. **c** Quantification of relative invasion and migration of images shown in **a** and **b**. Scatter dot plot, median with range (n = 03). ***P = 0.001; ****P < 0.0001. **d** Percent cell viability of Kyse150 and TE-1 cells 24 h after being transfected with either a control or MIR203A/5P mimic treated with indicated doses of 5-FU. Each replicate has been shown in a staggered plot (n = 03). ***P = 0.001; ****P < 0.0001

Given that Snail1 has been shown to be involved in EMT and metastatic progression in EC by transcriptionally lowering the expression of epithelial cell marker E-cadherin [9], we next investigated the effect of increasing miR-203a-5p expression levels in Kyse150 and TE-1 cells on *in vitro* invasion and migration, two critical functional readouts of EMT [7]. Increase in miR-203a-5p expression in Kyse150 cells downregulated *in vitro* invasion (Fig. 5a, c; P = 0.001) and migration (Fig. 5b, c; P < 0.0001) by 22.14 ± 2.83 and 37.02 ± 2.39 folds, respectively. Similar results were obtained in the TE-1 cells (invasion: 28.31 ± 3.19 folds, P < 0.0001; migration: 49.83 ± 3.34 folds, P < 0.0001) (Fig. 5a–c).

EMT is also characterized by increased chemoresistance of cancer cells as they metastasize to secondary sites. Hence, we next determined chemoresistance of control and MIR203A/5P mimic transfected Kyse150 and TE-1 cells to increasing concentration (0–50 µM) of 5-FU. The IC_50_ of 5-FU decreased from 18.23 ± 1.12 µM in control mimic to 4.01 ± 1.34 µM in MIR203A/5P mimic transfected Kyse150 cells (Fig. 5d; P = 0.001). Similarly, in the TE-1 cells the IC_50_ of 5-FU decreased from greater than 50 µM in control mimic to 5.11 ± 0.04 µM in MIR203A/5P mimic transfected cells (Fig. 5d; P < 0.0001). Cumulatively, the results show that modulating miR-203a-5p expression in EC cells impact Snail1 protein stability by decreasing USP26, which in turn inhibits the pro-metastatic functions of Snail1 in these cells.

### Suppression of miR-203-mediated regulation of USP26 is required for metastatic progression of EC cells

**Fig. 6:**
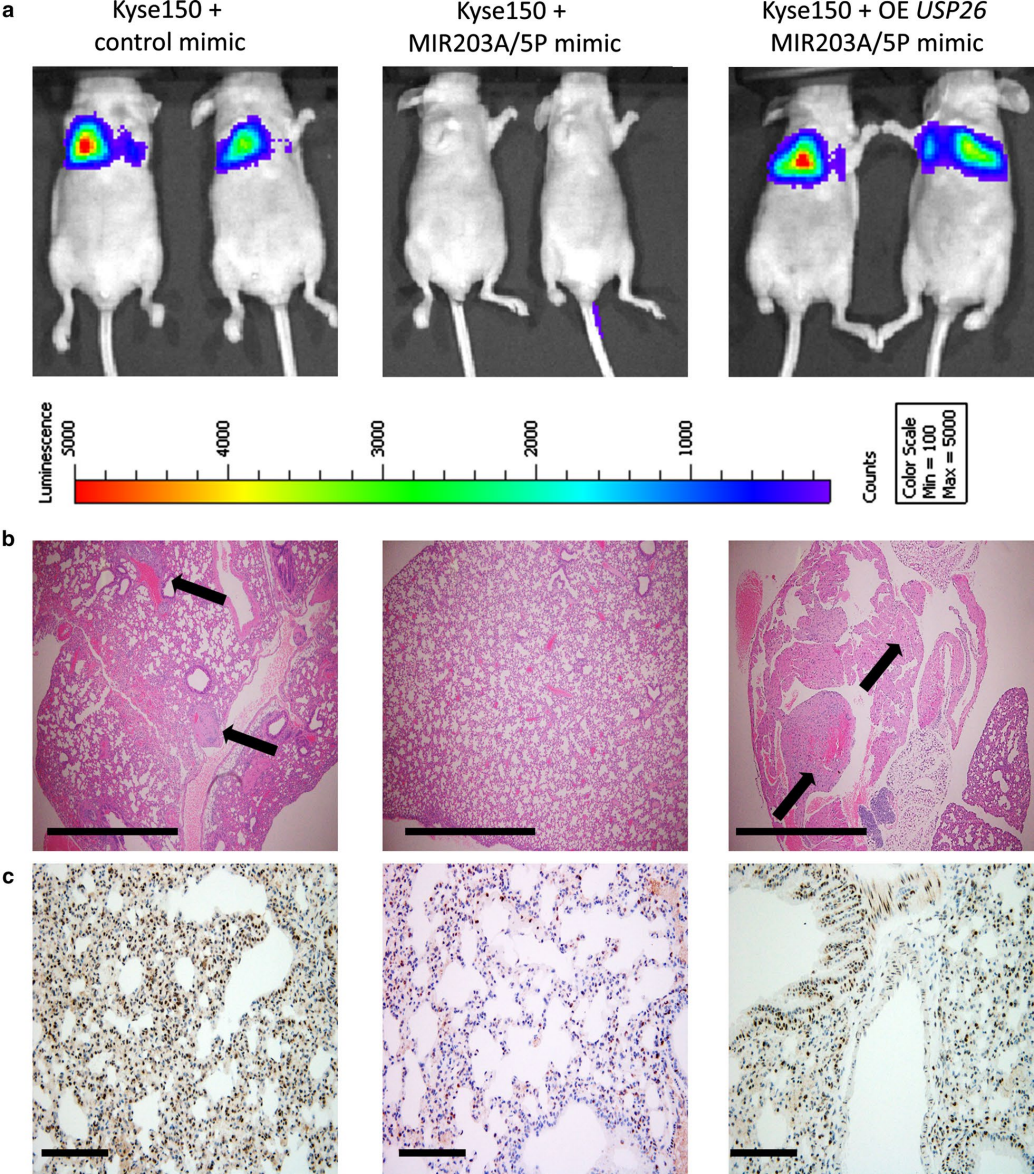
Suppression of miR-203-mediated regulation of USP26 is required for metastatic progression of EC cells. Xenograft model of experimental metastasis was established by lateral tail vein injection of parental Kyse150 cells or Kyse150 cells stably overexpressing *USP26* into BALB/c nude mice (n = 10 and 5 mice, respectively). The mice injected with parental Kyse150 cells were randomly divided into two groups – control mimic (n = 05) (*left panels*), and MIR203A/5P mimic (n = 05) (*middle panels*), where the mice injected with Kyse150 cells overexpressing *USP26* received MIR203A/5P mimic (*right panels*). The incidence of metastasis was measured by luciferin injection and bioluminescence imaging. Shown are representative animals from each group after 6 weeks (**a**). Mice were euthanized at the end of 6 weeks and the lungs from each group of experimental animals were surgically excised, fixed overnight in 10% buffered formalin. Representative images of hematoxylin and eosin (H and E) staining of the lungs are shown (**b**). Black arrows indicate micrometastasis. Scale bar, 100 µm. **c** Representative images of IHC staining for USP26 protein in each experimental group. Scale bar, 30 µm

We next wanted to determine if the in vitro findings of miR-203-mediated regulation of USP26 and pro-metastatic functions will hold in in vivo mice models of metastasis. Mice injected with parental Kyse150 cells had widespread lung metastasis after 6 weeks, as evident by bioluminescence signal (Fig. 6a, *left panel*) and micrometastatic lung lesions (Fig. 6b, *left panel*). IHC staining of lung lesions also revealed robust staining for USP26 in these mice (Fig. 6c, *left panel*). However, when these mice were injected with MIR203A/5P mimic every alternate day, it significantly decreased lung metastasis (Fig. 6a, b, *middle panels*). IHC staining also revealed downregulation of USP26 expression in the lung (Fig. 6c, *middle panel*). Given that miR-203 has many targets, we next wanted to confirm that its regulation of USP26 is indeed important for metastasis of EC cells. Hence, Kyse150 cells were stably transduced with a lentivirus expressing *USP26* and these cells were then injected via tail vein in the mice. The mice in these groups were also injected on alternate day with MIR203A/5P mimic. Overexpression of *USP26* reversed the inhibition of lung metastasis by MIR203A/5P mimic (Fig. 6a, b, *right panels*). As expected, robust expression of USP26 was seen in the lung lesions (Fig. 6c, *right panel*) in these groups of mice. Taken together these results corroborated our in vitro results and confirmed that deregulation of miR-203-mediated downregulation of USP26 is important for metastatic progression of EC.

## Discussion

Our study provides evidence that decreased expression of miR-203a-5p along with increased mRNA and protein expressions of Snail1 can be used as molecular markers of EC. Expression of USP26 protein, but not mRNA, is also increased in the context of EC cell lines. It remains to be determined if USP26 protein is also increased in EC patient samples. Our results also indicate the therapeutic potential of strategies to upregulate miR-203a-5p expression in EC cells. Obviously, *USP26* is not the only target of miR-203a-5p. In the context of EC, miR-203a’s function has been to inhibit β-catenin signaling, cell migration and invasion via directly inhibiting *KIF5C* expression in turn potentiating the antitumor activities of the downstream protein, Axin2 [24].

Different targets of miR-203a have been defined in the context of other cancers, like *RAB22A* (encoding Ras-related protein Rab-22A) and *BIRC5* (encoding survivin) in osteosarcoma [27, 28]. Hence, gene expression analysis post-miR-203A-5p mimic transfection in EC cells and miR-203-5p inhibitor transfection in HET-1A cells along with genome wide reporter assays will be required to identify the whole spectrum of genes that are being targeted directly by miR-203 in the context of EC. Once identified, gene ontology and functional analysis can be performed to identify major targets. Such studies will lead to pre-clinical therapeutic studies where miR-203 expression or its downstream targets will function as novel candidates. MiR-203 functions as a tumor suppressor in different cancer types [29–36]. Hence, it also needs to be determined if the downstream targets of miR-203 are similar or context dependent in different cancer types.

The other important thing that needs to be investigated is what regulates miR-203 expression. It has been shown that in esophageal squamous cancer cells, epidermal growth factor-induced truncated CCAAT-enhancer-binding protein β (C/EBPβ) LIP isoform transcriptionally downregulates miR-203 expression by directly interacting with a conserved distal regulatory element upstream of the miR-203 locus [37]. Also, in the context of esophageal cancer cells, it has been shown that cisplatin-induced E2F1 transactivates miR-203 by directly binding to its promoter [38]. More clarity is needed as to the precise mechanisms that regulate miR-203 expression in EC cells.

Finally, it is not surprising that given the importance of Snail1 protein in metastatic progression, expression of Snail1 is regulated at different stages of gene expression. Even USP26 is one of many DUBs that seem to stabilize Snail1 [14–18]. It remains to be determined if all the DUBs are being targeted by miR-203. If not, then it is important to define why just targeting USP26 by ectopic increase in miR-203 is enough to destabilize Snail1 protein. One possibility is that the DUBs are regulated or function as a group and when one member goes missing the entire protective mechanism falls apart.

## Conclusions

In summary, our study provides evidence that downregulation of miR-203 favors pro-metastatic behavior in EC cells by derepressing USP26 and stabilizing Snail1 protein. Our results add to the evidence highlighting miR-203 as an important biomarker of disease progression in EC and also highlight the potential of therapeutic benefits of upregulation of miR-203 in inhibiting or delaying metastatic progression in EC.

## Data Availability

All data generated or analysed during this study are included in this published article.

## Abbreviations

EC: Esophageal cancer
miRNA: microRNA
EMT: Epithelial to mesenchymal transition
DUBs: deubiquitinating enzymes
5-FU: 5-fluorouracil
